# Precritical State Transition Dynamics in the Attractor Landscape of a Molecular Interaction Network Underlying Colorectal Tumorigenesis

**DOI:** 10.1371/journal.pone.0140172

**Published:** 2015-10-06

**Authors:** Hyunho Chu, Daewon Lee, Kwang-Hyun Cho

**Affiliations:** Department of Bio and Brain Engineering, Korea Advanced Institute of Science and Technology (KAIST), Daejeon, Korea; University of Rome Tor Vergata, ITALY

## Abstract

From the perspective of systems science, tumorigenesis can be hypothesized as a critical transition (an abrupt shift from one state to another) between proliferative and apoptotic attractors on the state space of a molecular interaction network, for which an attractor is defined as a stable state to which all initial states ultimately converge, and the region of convergence is called the basin of attraction. Before the critical transition, a cellular state might transit between the basin of attraction for an apoptotic attractor and that for a proliferative attractor due to the noise induced by the inherent stochasticity in molecular interactions. Such a flickering state transition (state transition between the basins of attraction for alternative attractors from the impact of noise) would become more frequent as the cellular state approaches near the boundary of the basin of attraction, which can increase the variation in the estimate of the respective basin size. To investigate this for colorectal tumorigenesis, we have constructed a stochastic Boolean network model of the molecular interaction network that contains an important set of proteins known to be involved in cancer. In particular, we considered 100 representative sequences of 20 gene mutations that drive colorectal tumorigenesis. We investigated the appearance of cancerous cells by examining the basin size of apoptotic, quiescent, and proliferative attractors along with the sequential accumulation of gene mutations during colorectal tumorigenesis. We introduced a measure to detect the flickering state transition as the variation in the estimate of the basin sizes for three-phenotype attractors from the impact of noise. Interestingly, we found that this measure abruptly increases before a cell becomes cancerous during colorectal tumorigenesis in most of the gene mutation sequences under a certain level of stochastic noise. This suggests that a frequent flickering state transition can be a precritical phenomenon of colorectal tumorigenesis.

## Introduction

Cancer is a genetic disease driven by the accumulation of genetic mutations [1–3]. Genetic mutations lead to a cell undergoing suppressed cell death and uncontrolled cell proliferation, which are hall marks of cancer [4, 5]. From the view of systems science, tumorigenesis can be hypothesized as a critical transition between proliferative and apoptotic attractors upon the state space of a molecular interaction network, where an attractor is defined as a stable state to which all initial states ultimately converge, and the region of convergence is called the basin of attraction [6–9]. Creixell *et al*. [6] reviewed that the state space of dynamic cellular networks can be represented as attractor landscapes, where stable steady states (attractors) and unstable steady states are represented as valleys and mountains, respectively. They explained that cells are constantly navigating this attractor landscape and are pushed from one state to another by intracellular or different environmental cues, to drive biological decision processes. The sequential accumulation of genetic mutations may reshape the attractor landscape so that a cell is frequently attracted to proliferative attractors, resulting in tumorigenesis.

**Fig 1:**
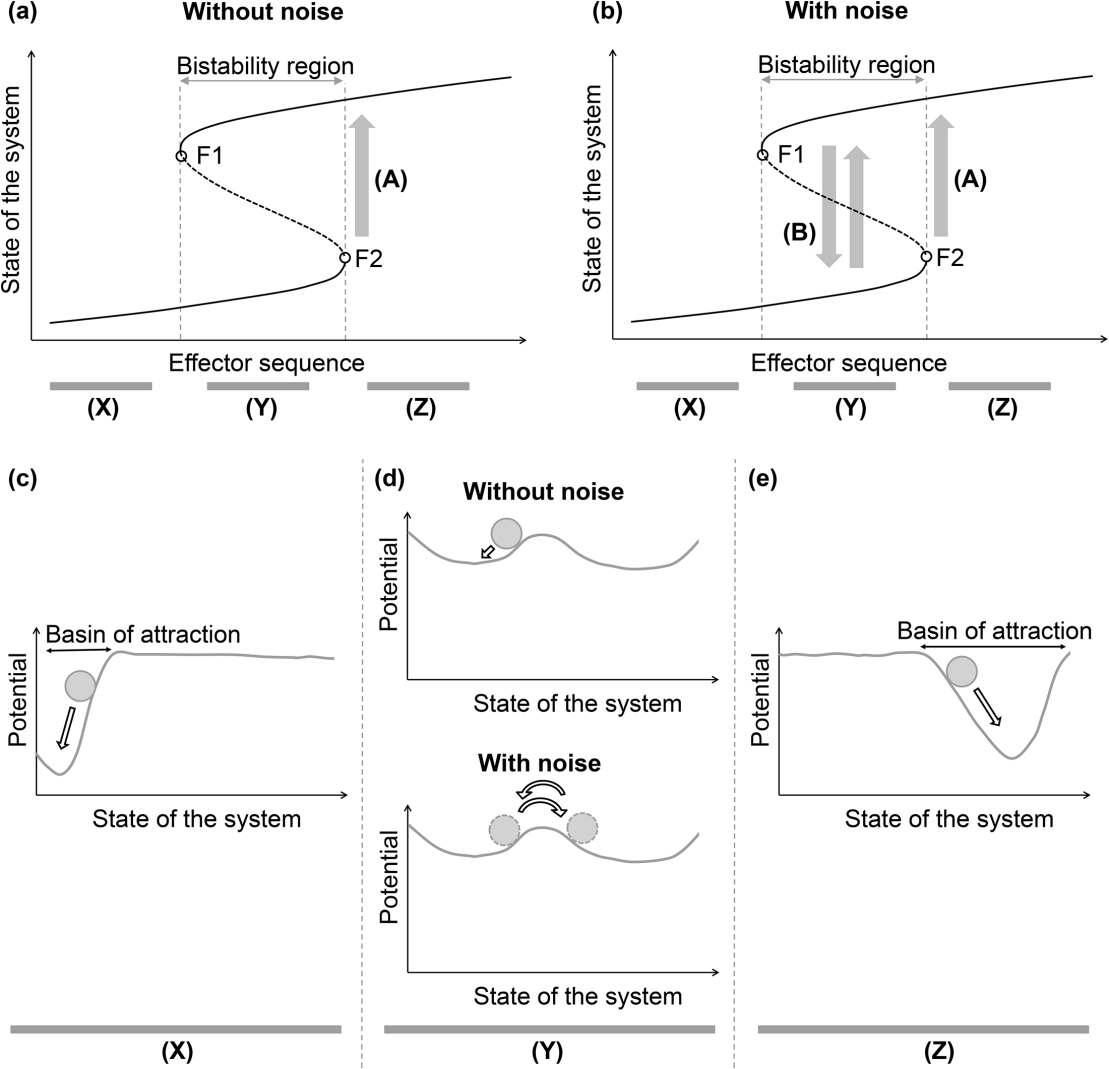
Flickering state transition before a critical transition in attractor dynamics. (a) and (b) represent critical transitions without and with noise in the attractor dynamics, respectively. The x-axis represents the effector sequence, and the y-axis denotes the state of the system. A solid line indicates an attractor, and the dotted line between the two solid lines represents an unstable state. A critical transition to an alternative attractor state (A) occurs at a bifurcation point (F1 or F2). Effectors in (a) and (b) are factors changing the attractor landscape. (c), (d), and (e) indicate the attractor landscapes reflecting the stability properties of the system in the region of (X), (Y), and (Z), respectively. Because a potential on the y-axis is inversely related to the steady state probability of its state, the dynamics tends to converge to a state with lower potential. A ball (grey circle) represents the current state and its potential. (d) In the region of (Y), the ball jumps back and forth between alternative basins of attraction from the impact of noise, namely the flickering state transition ((B) in Fig 1(b)). Such a flickering state transition increases the variation in the estimate of the basin sizes (the sizes of the basin of attraction) for the attractors.

Scheffer *et al*. explained that, in a wide range of natural systems, there are generic early-warning signals before critical transitions, regardless of the differences in the details of each system [10, 11]. Based on attractor dynamics, they suggested that “flickering to an alternative state” could be one of the early-warning signals before critical transitions in stochastic systems [10–12]. Fig 1 shows a flickering state transition before a critical transition in attractor dynamics. The solid line in Fig 1(a) and 1(b) represents an attractor state. The dotted line in Fig 1(a) and 1(b) indicates unstable states and the boundary between two basins of attraction for two attractor states. Effectors in Fig 1(a) and 1(b) are factors changing the attractor landscape. Fig 1(c), 1(d) and 1(e) indicates the attractor landscapes reflecting the stability properties of the system in the region of (X), (Y), and (Z), respectively. Because lower potential means a higher steady state probability, a state spontaneously transits to another state with a lower potential. A critical transition to an alternative state ((A) in Fig 1(a) and 1(b)) occurs at a bifurcation point (F1 or F2 in Fig 1(a) and 1(b)). The frequent flickering state transition is an observable phenomenon in the vicinity of a bifurcation point in a noisy environment. In Fig 1, in the region of (X) and (Z), the attractor of the state of a system is strong enough to capture the system from the impact of noise, and a small variance appears in the estimate of the basin sizes of the attractors from the impact of noise. However, as the system enters the bistability region of (Y) before settling more permanently into an alternative state, the attractor of the state of the system is too weak to capture the system from the impact of noise, and the impact from noise causes the state of the system to frequently transit ((B) in Fig 1(b)) between the basins of attraction for the two alternative attractors. Such a flickering state transition leads the state within the basin of attraction for an attractor to converge into another attractor and increases the variation in the estimate of the basin sizes (the sizes of the basin of attraction) for the attractors. The frequent flickering state transition in a system can be considered a warning that the system has left the stable operating state space.

In colorectal cancer studies, gene mutations leading to colorectal tumorigenesis can correspond to effectors changing the attractor landscape of a cell system. The sequential accumulation of gene mutations can make the system of a normal cell enter the bistability region before the system settles more permanently into a colorectal cancer cell. We investigated the existence of a flickering state transition during colorectal tumorigenesis, resulting from some noise induced by the inherent stochasticity in molecular interactions in cells [13–16], with a stochastic Boolean network modeling and simulation approach.

## Results and Discussion

Under various levels of noise intensity, we investigated the presence of a flickering state transition during colorectal tumorigenesis in a stochastic Boolean network model of a cell. The model consists of 96 nodes and 246 edges. Ninety-one nodes represent an important subset of proteins involved in cancer, and 5 input nodes represent carcinogens, growth factors, nutrient supply, growth suppressors, and hypoxia that give distinct environmental stimuli and stresses to a cell (i.e., 2^5^ combinations for environmental conditions). The values of the nodes are synchronously updated following the rules described in the S1 Text. A noise intensity parameter N_I_ is introduced so that the state of each node is randomly re-assigned at every time step: after updating the values of the nodes, the state of each node is flipped with the probability of N_I_. Colorectal tumorigenesis is driven by the sequential accumulation of 20 gene mutations with 100 representative sequences (S1 Table). In the constructed model, a mutation activating an oncogene is represented as a permanent activation of the corresponding protein node, and a mutation inactivating a tumor suppressor gene is expressed as a permanent inactivation of the corresponding protein node. For every occurrence of a gene mutation, 10,000 initial states for each environmental condition (i.e., a total of 320,000 initial states for 2^5^ combinations of environmental conditions) were randomly selected and analyzed to get the fractions of the basin size of attractors. With a random procedure of N_I_≠0, there was no fixed point or limit cyclic attractors. Therefore, we tested all the randomly selected initial states, running them for 1,100 time steps, expecting to reach clean (N_I_ = 0) or noisy (N_I_≠0) attractors until 1,000 time steps in the model (with N_I_ = 0, all the randomly selected initial states converge to fixed point or limit cyclic attractors within about 30 time steps). We decided the phenotype of an attractor in the model, considering the states of 91 nodes except for the 5 environment nodes from 1,001 to 1,100 time steps in the model. The apoptosis phenotype is given when the activity (% of ‘1’s in a given time window) of the apoptosis node in the model is more than 80%. The proliferative phenotype is given when cyclins are correctly activated along the cell cycle (cyclin D → cyclin E → cyclin A → cyclin B) at least 4 times (it avoids the decision of the proliferative phenotype by a temporary increase in the frequency of the cell-cycle activation from noise, and cyclins are correctly activated along the cell cycle 14 times with N_I_ = 0). The quiescent phenotype is given when both the proliferative phenotype and the apoptosis phenotype are not given.

**Fig 2:**
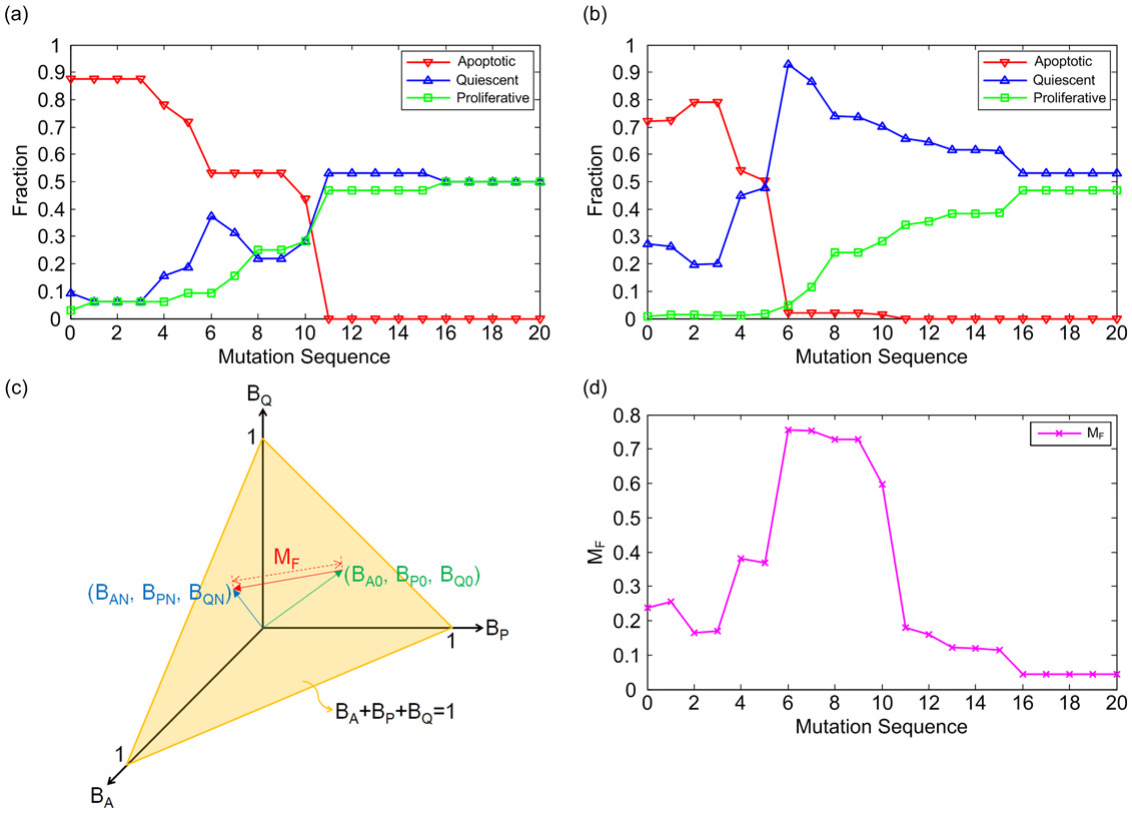
Flickering state transition during colorectal tumorigenesis in the conditions of N_I_ = 0.03. Colorectal tumorigenesis is driven by the sequential accumulation of 20 gene mutations (the sequence of No. 73 in the S1 Table). Zero on the x-axis means no mutation. (a) and (b) the fraction of the initial states converging into the apoptotic, proliferative or quiescent attractors for 320,000 initial states at every gene mutation with N_I_ = 0 and 0.03, respectively. (c) A graphical representation of M_F_ (Eq (1)) to check the flickering state transition. B_A_, B_P_, and B_Q_ represent the fractions of the basin sizes for the apoptotic, proliferative, and quiescent attractors, respectively. B_A0_, B_P0_, and B_Q0_ express the fractions of the basin sizes for the apoptotic, proliferative, and quiescent attractors in the absence of noise, respectively, and B_AN_, B_PN_, and B_QN_ are the fraction of the basin sizes for the apoptotic, proliferative, and quiescent attractors in the presence of noise, respectively. (d) M_F_ at every mutation occurrence for N_I_ = 0.03, as a result of Fig 2(a) and (b).

In Fig 2, the simulation results show the appearance of the flickering state transition during colorectal tumorigenesis in the conditions of N_I_ = 0.03. Colorectal tumorigenesis is driven by the sequential accumulation of 20 gene mutations (the sequence of No. 73 in the S1 Table). Fig 2(a) and 2(b) shows the fraction of the initial states converging into apoptotic, proliferative or quiescent attractors for 320,000 initial states at every gene mutation with N_I_ = 0 and 0.03, respectively. The fraction of the initial states converging into attractors with a particular phenotype represents the estimate of the ratio of the basin size for attractors with this phenotype to the sum of the basin sizes for the three-phenotype attractors. To check the flickering state transition, we have introduced a measure, M_F_, to measure how many states switch back and forth between the basins of attraction for the proliferative, apoptotic, and quiescent attractors from the impact of noise:

$${\text{M}}_{\text{F}}=\sqrt{{\left({\text{B}}_{\text{AN}}-{\text{B}}_{\text{A0}}\right)}^{2}+{\left({\text{B}}_{\text{PN}}-{\text{B}}_{\text{P0}}\right)}^{2}+{\left({\text{B}}_{\text{QN}}-{\text{B}}_{\text{Q0}}\right)}^{2}},$$ (1)

where B_A0_, B_P0_, and B_Q0_ are the fractions of the basin sizes for the apoptotic, proliferative, and quiescent attractors in the absence of noise, respectively, and B_AN_, B_PN_, and B_QN_ are the fractions of the basin sizes for the apoptotic, proliferative, and quiescent attractors in the presence of noise, respectively. Fig 2(c) shows a graphical representation of M_F_. M_F_ reflects the variation in the estimate of the basin sizes of the three-phenotype attractors due to the transitions of states between the basins of attraction for the three-phenotype attractors from the impact of noise. M_F_ appears, as a state within the basin of attraction for an attractor with a phenotype converges into another attractor with a different phenotype, due to the transition of the state between the basins of attraction for these two attractors from the impact of noise. M_F_ will increase as the transitions of the states happen more actively between the basins of attraction for the three-phenotype attractors from the impact of noise. Fig 2(d) shows the M_F_ at every mutation occurrence for N_I_ = 0.03 as a result of Fig 2(a) and 2(b). In Fig 2(a) and 2(b), the cancerous state occurs at the 11^th^ mutation occurrence (the cancerous state means a state where a cell shows uncontrolled proliferation which is believed to be malignant, and in this study the cancerous state is defined as the state in which the fraction of the basin size for the proliferative attractors is more than 0.35 and that of the apoptotic attractors is less than 0.05). In Fig 2(d), the M_F_ is relatively low until the 5^th^ mutation occurrence, and increases sharply at the 6^th^ mutation occurrence, finally decreasing as the cancerous state begins, indicating the existence of more frequent flickering state transitions before the critical transition to the cancerous state. Looking at the implications, a cellular state transits between the basins of attraction for the three-phenotype attractors from some noise induced by the inherent stochasticity in molecular interactions [13–16]. Such flickering state transitions would be more frequent before a cell becomes cancerous during colorectal tumorigenesis, indicating that the cell has left its stable operating state space: the attractor landscape of the cell has been deformed, as the potentials of existing attractors become higher (becoming unstable from the impact of noise) or as new attractors begin to emerge.

**Fig 3:**
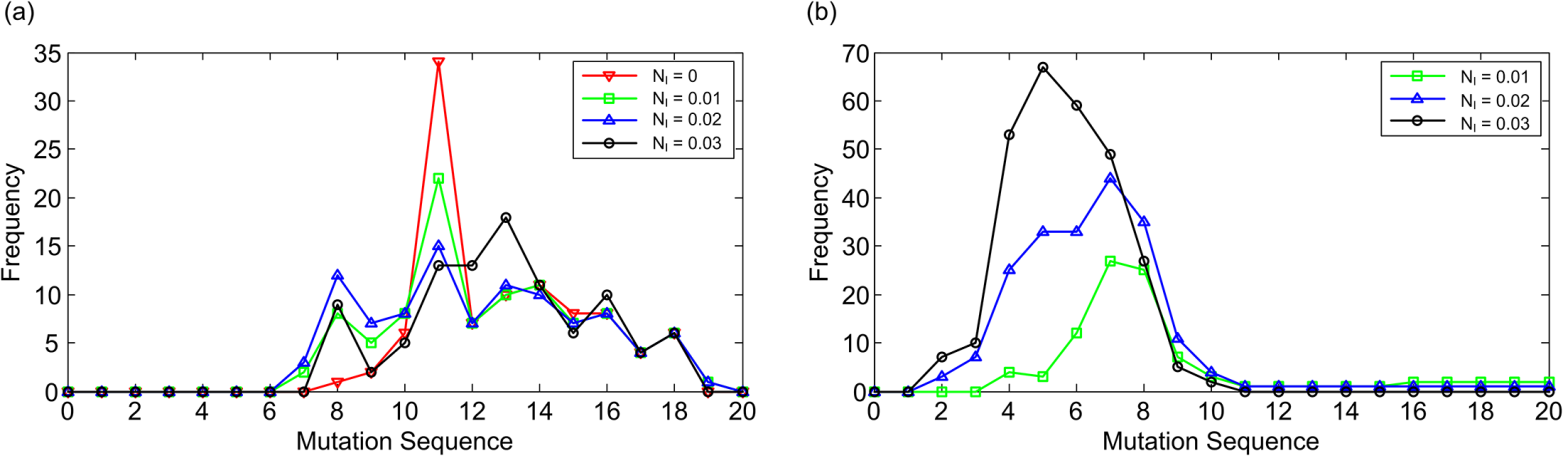
Generality of the more frequent flickering state transition before developing into colorectal cancer. Colorectal tumorigenesis is driven by the sequential accumulation of 20 gene mutations for 100 representative sequences for 20 gene mutations that drive colorectal tumorigenesis (S1 Table). Zero on the x-axis means no mutation. (a) The frequency distribution of the occurrence point of the cancerous state along with the sequences that drove the cancerous state for the various levels of noise intensity. For the noise intensities of 0, 0.01, 0.02, and 0.03, the cancerous state occurs in 97, 99, 99, and 97 sequences of the 100 sequences, respectively (Table 1). (b) The frequency distribution of the M_F_ greater than the M_FTH_ at every mutation occurrence along with the sequences that drove the cancerous state for the various levels of noise intensity. We defined the upper threshold of M_F_ (M_FTH_) to investigate whether the flickering state transition becomes more frequent in the attractor landscape. For the noise intensities of 0.01, 0.02, and 0.03, an M_F_ greater than the M_FTH_ appears in 46, 80, and 81 sequences of 99, 99, and 97 sequences that drove the cancerous state, respectively (Table 1). The y-axis indicates how many sequences among the sequences that drove the cancerous state have an M_F_ greater than the M_FTH_ at a particular mutation occurrence.

To investigate the generality of the precancerous occurrence of the more frequent flickering state transition during colorectal tumorigenesis (the precancerous occurrence means the occurrence before a cell becomes cancerous), we considered 100 representative sequences for 20 gene mutations to drive colorectal tumorigenesis (S1 Table), for which all gene mutations occur toward colorectal tumorigenesis (tumor suppressor genes and oncogenes mutate to permanently inactivate and activate the corresponding protein nodes, respectively). Fig 3(a) and 3(b) statistically shows when the cancerous state occurs, and M_F_ significantly increases along with these 100 sequences for various levels of noise intensity. Fig 3(a) shows the frequency distribution of the occurrence point for the cancerous state along with the sequences that drove the cancerous state for the various levels of noise intensity. For the noise intensities of 0, 0.01, 0.02, and 0.03, the cancerous state occurs in 97, 99, 99, and 97 sequences of the 100 sequences, respectively (Table 1). For the sequences that have driven the cancerous state, we investigated when a significant increase of M_F_ appears during colorectal tumorigenesis for the various levels of noise intensity. We experimentally set the upper threshold of M_F_ as follows: M_FTH_ = M_F0_+0.24 (M_FTH_: upper threshold of M_F_, M_F0_: M_F_ at no mutation occurrence). It lets M_FTH_ exceed the sum of the mean and standard deviation of the values of M_F_ greater than the M_F0_, when the values of M_F_ are measured along with random gene mutations. For random gene mutations, we considered 100 sequences for 20 random gene mutations for which all the genes were randomly selected, and the mutation states were randomly determined regardless of tumorigenesis (S2 Table). When the M_F_ was greater than M_FTH_, we decided that the M_F_ has increased more significantly than that which can be produced by random gene mutations, and the flickering state transition becomes more frequent in the attractor landscape. Fig 3(b) shows the frequency distribution of the M_F_ greater than the M_FTH_ at every mutation occurrence along with the sequences that drove the cancerous state for the various levels of noise intensity. For the noise intensities of 0.01, 0.02, and 0.03, an M_F_ greater than the M_FTH_ appears in 46, 80, and 81 sequences of the 99, 99, and 97 sequences that drove the cancerous state, respectively (Table 1). The y-axis indicates how many sequences among the sequences that drove the cancerous state have an M_F_ greater than the M_FTH_ at a particular mutation occurrence. An M_F_ greater than the M_FTH_ generally appears from the 4^th^ mutation occurrence to the 8^th^ mutation occurrence before the occurrence of the cancerous state shown in Fig 3(a). We statistically estimated the difference between two distributions for the occurrence point of the cancerous state and an M_F_ greater than the M_FTH_ along with the following sequences: the sequences that drove both the cancerous state and an M_F_ greater than the M_FTH_ are presented in S1 Table. By two-sample Kolmogorov-Smirnov test [17, 18], for N_I_ = 0.01, 0.02, and 0.03, the p-values were 2.29×10^−15^, 8.77×10^−30^, and 9.48×10^−47^, respectively. These results support the generality that the flickering state transition becomes more frequent before a cell becomes cancerous during colorectal tumorigenesis. To verify the possibility that the flickering state transition becomes more frequent by chance, 100 sequences for 20 random gene mutations regardless of tumorigenesis (S2 Table) were implemented in the stochastic Boolean network model of a cell. All 100 sequences did not drive the cancerous state (Table 1). This result means that the cancerous state hardly occurs by random mutations because the biological system is very robust. For the noise intensities of 0.01, 0.02, and 0.03, an M_F_ greater than the M_FTH_ appears in 3, 22, 22 sequences of these 100 sequences, respectively (Table 1). We statistically evaluated the difference between two distributions for an M_F_ greater than the M_FTH_ along with the following two sequence sets: the sequences that drove the cancerous state in S1 Table, and the 100 sequences listed in S2 Table. By two-sample Kolmogorov-Smirnov test, for N_I_ = 0.01, 0.02, and 0.03, the p-values were 2.42×10^−6^, 1.37×10^−14^, and 6.54×10^−19^, respectively. It implies that an M_F_ greater than the M_FTH_, along with the random mutation sequences, does not have precancerous characteristics. In summary, the gene mutation sequence that drove the colorectal cancer has a superior chance to result in a significant increase of M_F_ before the occurrence of the cancerous state, compared to the random gene mutation sequence. This suggests that a more frequent flickering state transition can be a precritical phenomenon in the attractor landscape of a molecular interaction network underlying colorectal tumorigenesis.

**Table 1:** The number of sequences for gene mutations that resulted in an M_F_ greater than the M_FTH_.

	100 sequences for colorectal tumorigenesis (S1 Table)				100 sequences regardless of tumorigenesis (S2 Table)			
	A	B	C	D	A	B	C	D
**N**_**I**_ = **0**	97		3		0		100	
**N**_**I**_ = **0.01**	99	46	1	0	0	0	100	3
**N**_**I**_ = **0.02**	99	80	1	0	0	0	100	22
**N**_**I**_ = **0.03**	97	81	3	2	0	0	100	22

A: the number of sequences that drove the cancerous state.

B: the number of sequences that drove the cancerous state and had an M_F_ greater than the M_FTH_.

C: the number of sequences that did not drive the cancerous state.

D: the number of sequences that did not drive the cancerous state but had an M_F_ greater than the M_FTH_.

In this study, we described that the interplay of genetic mutations and noise drive tumor progression. The accumulation of genetic mutations during colorectal tumorigenesis plays a mechanistic role of reshaping the attractor landscape (the basin sizes of proliferative and apoptotic attractors increases and decreases, respectively), and it increases the chance of noise-induced transitions toward cancerous states. There is a dichotomy of views on the perturbations that drive tumor progression: genetic and non-genetic perturbations [19, 20]. From the perspective of genetic perturbations, tumor progression is driven by the accumulation of genetic mutations. On the other hand, from the perspective of non-genetic perturbations, tumor progression is promoted by the perturbation-induced state transition to cancer attractors, regardless of mutations. Our study presents the confluence of genetic and non-genetic perturbations (mutations and noise) to drive tumor progression.

## Conclusions

Under a noisy environment, the frequent flickering state transition occurs before a critical transition to an alternative state; from the impact of noise, the state of a system frequently switches back and forth between the basins of attraction for alternative attractors, and it increases the variation in the estimate of the respective basin sizes. In this study, we constructed a stochastic Boolean network model of a molecular interaction network that contains an important set of proteins known to be involved in cancer, and investigated the existence of a flickering state transition, driving colorectal tumorigenesis by the sequential accumulation of gene mutations. As a result, we have found that the flickering state transition is more frequent before a cell becomes cancerous during colorectal tumorigenesis for a certain level of noise intensity, implying that the cell has left its stable operating state space. This finding provides significant insight into the relationship between a cell system and its stability to noise. In addition, this finding can only be revealed when considering the interplay between the structural constraints imposed by the underlying molecular interaction network and the effect of genetic and non-genetic perturbations (mutation and noise). Furthermore, if the occurrences of other diseases are considered critical transitions from normal states to diseases states in the attractor landscape, we expect that their flickering state transitions during pathogenesis could also provide the precritical phenomenon of disease occurrences.

## Methods

### Boolean modeling of the molecular interaction network related to colorectal tumorigenesis

For the modeling of colorectal tumorigenesis, we used the cancer Boolean network model proposed by Fumiã *et al*. [21]. Because we were mainly concerned with the overall dynamic properties and stability of the network to noise, we used a simplified dynamics on the network, which treats the nodes and arrows as logic-like operations [22, 23]. Fumiã *et al*. explained that the model integrated the main signaling pathways involved in cancer and was constructed based on currently known literature [24, 25] and the molecular interaction network reported in the KEGG database [26]. In particular, subgraphs of the PI3K-AKT, mTOR, MAPK, HIF1, TGF-beta, WNT, NF-Kb, TNF, cell cycle, p53, and apoptosis KEGG pathways were included in the model [27–35]. Randomly sampled initial states converge into one of 62 attractors (36 fixed points and 26 limit cycles), and these attractors are classified into three groups characterized by specific phenotypes: apoptotic, proliferative, and quiescent. Fumiã *et al*. proved the validity of the model, showing that the predictions of the three-phenotype attractors from the model under the combinations of 5 environmental conditions are consistent with experimental results reported in [36–38]. S1 Fig shows this network consisting of 96 nodes and 246 edges.

To numerically analyze the dynamical behavior of the cell system under the noise induced by the inherent stochasticity in molecular interactions [13–16], we modified the original model to have a stochastic noise procedure. To this aim, we defined a random procedure for changing the state of each node at every time step. A noise intensity parameter N_I_ is introduced so that the state of each node is randomly re-assigned at every time step as follows [39, 40]:

$${\text{O}}_{\text{i}}(\text{t}+1)=\{\begin{array}{ll} \text{g}({\text{O}}_{\text{i}1},{\text{O}}_{\text{i}2},\ldots ,{\text{O}}_{\text{iP}(\text{i})}) & \text{with probability }1-{\text{N}}_{\text{I}}\\ 1-\text{g}({\text{O}}_{\text{i}1},{\text{O}}_{\text{i}2},\ldots ,{\text{O}}_{\text{iP(i)}}) & \text{with probability }{\text{N}}_{\text{I}} \end{array},$$ (2)

where O_i_ is the state of the i^th^ node of total M nodes: {O_i_}_i = 1,…, M_. The i^th^ node is connected to P(i) nodes of the network (0<P(i)≤M), which define the states of the regulators of the i^th^ node: {O_ij_}_j = 1,…, P(i)_. g(O_i1_, O_i2_,…, O_iP(i)_) represents the next state of the i^th^ node determined based on the logic statement on the current states of its regulators without a stochastic noise procedure. N_I_ is between 0 and ½. For N_I_ = 0, the dynamics are wholly deterministic and fully governed by the rules referred in the S1 Text, while for N_I_ = ½, they are wholly random.

### Sampling procedure

To get the fractions of the basin size of attractors, 10,000 initial states for each environmental condition (i.e., a total of 320,000 initial states for 2^5^ combinations of environmental conditions) were randomly sampled following a uniform distribution over the state space after having fixed the given mutations and the environmental conditions. In the sampling procedure, each of the 91 nodes except for the 5 input nodes has an independent probability of ½ to be in either the ON state or the OFF state (with the fixed input nodes, it ensures that sampling of each state for all 2^91^ states is uniformly made with the probability of (½)^91^). The random sampling of the appropriate number of initial states produces fairly robust estimates of the basin sizes. S3 Table shows the estimates of the basin sizes as the sampling size increases. Simulation results using the random sampling of 10,000, 100,000, and 1,000,000 initial states for each environmental condition (i.e., a total of 320,000, 3,200,000, and 32,000,000 initial states for 2^5^ combinations of environmental conditions, respectively) were compared, repeating the estimation procedure 3 times for each sample size, respectively. The results show that the random sampling of 10,000 initial states for each environmental condition is appropriate for these fairly robust estimates of the basin sizes. In the S2 Text, the relative effects on the observed variation in the estimate of the basin size by the genetic perturbation (mutation), the non-genetic perturbation (noise), and the sampling procedure used in this study are discussed.

### Gene mutation sequence

Colorectal tumorigenesis is driven by the sequential accumulation of somatic mutations in oncogenes and tumor suppressor genes [2, 41–44]. Fearon summarized the types of somatic mutations per gene in colorectal tumorigenesis: point mutation (nonsense, missense, and frameshift), gene amplification, allele loss, and deletion [42]. On average, colorectal cancer can have about 60 to 70 protein-altering mutations, of which about 3 to 7 may be driver gene mutations, and the remaining ones may be passenger gene mutations [1, 45]. A driver gene mutation directly or indirectly gives a selective growth advantage to a cell [46]. On the other hand, a passenger gene mutation does not increase the selective growth advantage of a cell. In this study, the number of gene mutations during colorectal tumorigenesis is defined as 20. In a sequence of 20 gene mutations, the number of driver gene mutations and additional gene mutations are defined as 5 and 15, respectively. They are sequentially accumulated to drive colorectal tumorigenesis in the constructed model. The 5 driver genes [24, 41, 42, 47, 48] and the order of their mutations [41, 42] for colorectal tumorigenesis are defined as APC, RAS, PTEN, SMAD, and p53. The 15 genes are randomly selected from the protein nodes in the constructed model. In particular, the RAF does not mutate because mutations of RAS and RAF rarely co-occur together in the same patient with colorectal cancer [49]. Because a tumor is initiated by a driver gene mutation [2] and colorectal tumorigenesis is mostly initiated by an APC mutation [41], an APC mutation is always added first, and the other 19 gene mutations are added in random order, maintaining the order of the driver gene mutations. We generated 100 representative sequences of 20 gene mutations to drive colorectal tumorigenesis (S1 Table).

## Supporting Information

S1 Fig Boolean network model for colorectal tumorigenesis. The model was proposed by Fumiã *et al*. [21]. It consists of 96 nodes and 246 edges. Ninety-one nodes represent an important subset of proteins involved in cancer, and 5 input nodes, represented as yellow circles, express carcinogens, growth factors, nutrient supply, growth suppressors, and hypoxia that give distinct environmental stimuli and stresses to a cell. Activating and inhibiting interactions between nodes are expressed by blue arrows and red lines with a bar, respectively.
(TIF)

S2 Fig The Euclidean distance between two vectors of (B_A_, B_P_, B_Q_), indicating the variation in the estimate of the basin size by the sampling procedure. (TIF)

S1 Table 100 sequences for 20 gene mutations that drive colorectal tumorigenesis. (XLSX)

S2 Table 100 sequences for 20 gene mutations for which all the genes were randomly selected and the mutations randomly occurred regardless of tumorigenesis. (XLSX)

S3 Table The estimate of the basin size according to the variation of the sample size. (XLSX)

S1 Text The update rules for the 96 nodes in the cancer Boolean network. (PDF)

S2 Text Relative effects on the observed variation in the estimate of the basin size by the mutation, the noise, and the sampling procedure. (PDF)
