# Supplementary figures and images for: Precritical State Transition Dynamics in the Attractor Landscape of a Molecular Interaction Network Underlying Colorectal Tumorigenesis
Source: PLoS One. 2015 Oct 6;10(10):e0140172. doi: 10.1371/journal.pone.0140172 (PMC4595005; doi:10.1371/journal.pone.0140172)

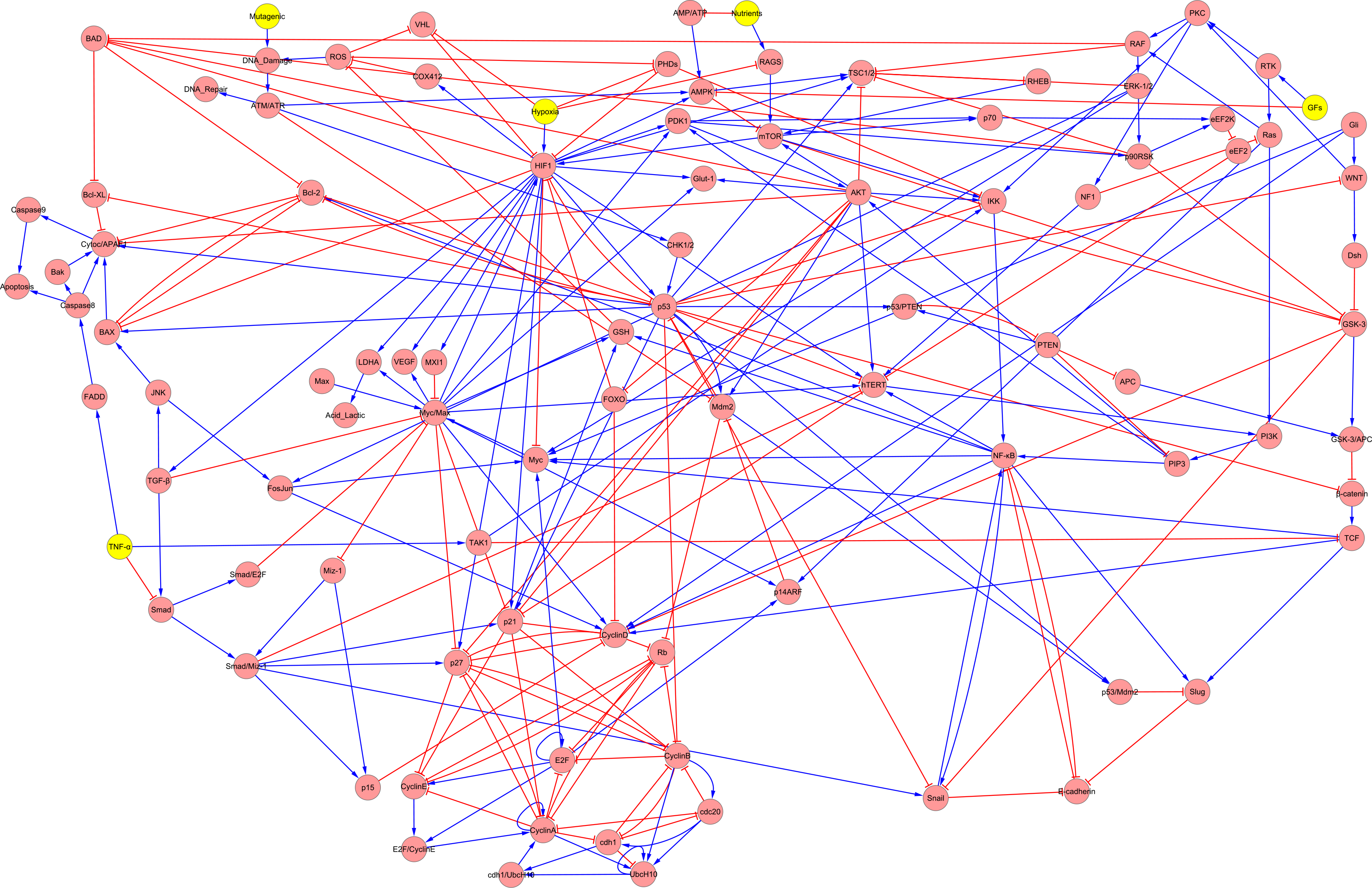

Supplement: S1 Fig — The model was proposed by Fumiã et al. [21]. It consists of 96 nodes and 246 edges. Ninety-one nodes represent an important subset of proteins involved in cancer, and 5 input nodes, represented as yellow circles, express carcinogens, growth factors, nutrient supply, growth suppressors, and hypoxia that give distinct environmental stimuli and stresses to a cell. Activating and inhibiting interactions between nodes are expressed by blue arrows and red lines with a bar, respectively. (TIF) [file pone.0140172.s001.tif]

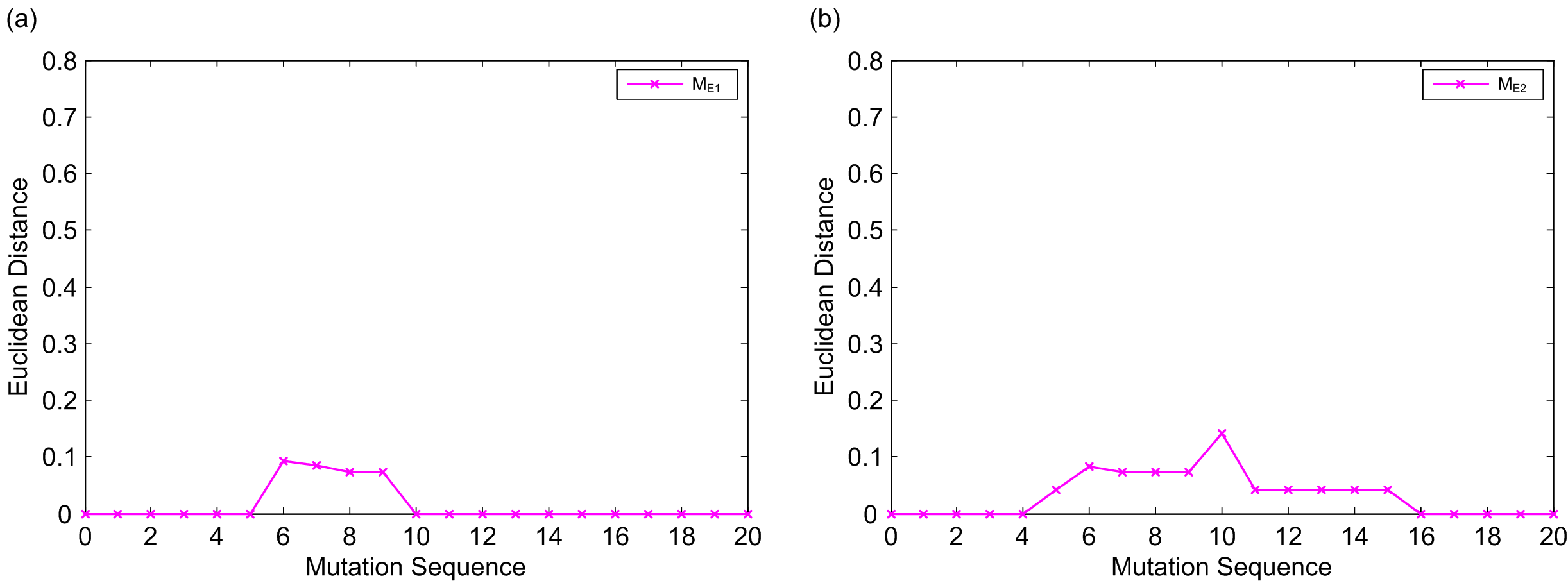

Supplement: S2 Fig — (TIF) [file pone.0140172.s002.tif]
