# Supplementary material for: Precritical State Transition Dynamics in the Attractor Landscape of a Molecular Interaction Network Underlying Colorectal Tumorigenesis
Source: PLoS One. 2015 Oct 6;10(10):e0140172. doi: 10.1371/journal.pone.0140172 (PMC4595005; doi:10.1371/journal.pone.0140172)
Supplement: S1 Text — (PDF) [file pone.0140172.s006.pdf]

S1 Text. The update rules for the 96 nodes in the cancer Boolean network

The definition of sgn function

$$\mathrm{sgn}(x) = \begin{cases} 0 & \text{if} \quad x \le 0 \\ 1 & \text{if} \quad x > 0 \end{cases}.$$

The general form of update rules

$$O_i(t+1) = \mathrm{sgn}\left(\sum_{j=1}^{P(i)} I_{ij} O_j(t) - T_i\right).$$

O_i is the state of the ith node of total M nodes: $\{O_i\}_{i=1,\dots,M}$. The ith node is connected to P(i) nodes of the network ($0<P(i)\le M$). I_{ij} represents the interaction strength from input j on protein i. The activation interaction strength is positive and the inhibition interaction strength is negative. T_i is the activation threshold of protein i.

The update rules for the 96 nodes in the cancer Boolean network

The network contains the subset of proteins in the PI3K-AKT, mTOR, MAPK, HIF1, TGF-beta, NF-kB, TNF, WNT, Rb-E2F, p53, and apoptosis pathways.

$O_{Mutagen}(t+1) = \text{input};$

$O_{GFs}(t+1) = \text{input};$

$O_{Nutrients}(t+1) = \text{input};$

$O_{TNFalpha}(t+1) = \text{input};$

$O_{Hypoxia}(t+1) = \text{input};$

$O_{Gli}(t+1) = \text{input};$

$O_{TGFbeta}(t+1) = \mathrm{sgn}[+O_{HIF1}(t)];$

$O_{DnaDamage}(t+1) = \mathrm{sgn}[+O_{Mutagen}(t) + O_{ROS}(t)];$

$O_{p53/Mdm2}(t+1) = \mathrm{sgn}[+O_{p53}(t) + O_{Mdm2}(t) - 1];$

$O_{AMP/ATP}(t+1) = \mathrm{sgn}[-O_{Nutrients}(t) + 1];$

$O_{NF1}(t+1) = \mathrm{sgn}[-O_{PKC}(t) + 1];$

$O_{PKC}(t+1) = \mathrm{sgn}[+O_{RTK}(t) + O_{WNT}(t)];$

$O_{RTK}(t+1) = \mathrm{sgn}[+O_{GFs}(t)];$

$O_{RAGS}(t+1) = \mathrm{sgn}[+O_{Nutrients}(t) - O_{Hypoxia}(t)];$

$O_{Ras}(t+1) = \mathrm{sgn}[-O_{NF1}(t) + O_{RTK}(t) + 1];$

$O_{PI3K}(t+1) = \mathrm{sgn}[+O_{Ras}(t) + O_{hTERT}(t)];$

$O_{PTEN}(t+1) = 1;$

$O_{PIP3}(t+1) = \mathrm{sgn}[+O_{PI3K}(t) - O_{PTEN}(t) - O_{p53/PTEN}(t) + 1];$

$$O_{PDK1}(t+1) = sgn[+O_{PIP3}(t) + O_{HIF1}(t) + O_{Myc/Max}(t)];$$

$$O_{IKK}(t+1) = sgn[+O_{PKC}(t) + O_{AKT}(t) + O_{mTOR}(t) - O_{PHDs}(t) - O_{p53}(t) + O_{TAK1}(t)];$$

$$O_{NF-kB}(t+1) = sgn[+O_{PIP3}(t) + 2O_{IKK}(t) - O_{E-cadh}(t) + O_{Snail}(t) - 1];$$

$$O_{RAF}(t+1) = sgn[+O_{PKC}(t) + O_{Ras}(t)];$$

$$O_{ERK}(t+1) = sgn[+O_{RAF}(t)];$$

$$O_{p90}(t+1) = sgn[+O_{PDK1}(t) + O_{ERK}(t)];$$

$$O_{AKT}(t+1) = sgn[+O_{PIP3}(t) + O_{PDK1}(t) - 1];$$

$$O_{WNT}(t+1) = sgn[-O_{p53}(t) + O_{Gli}(t)];$$

$$O_{Dsh}(t+1) = sgn[+O_{WNT}(t)];$$

$$O_{APC}(t+1) = sgn[+O_{PTEN}(t) + 1];$$

$$O_{GSK-3}(t+1) = sgn[-O_{p90}(t) - O_{AKT}(t) - O_{Dsh}(t) - O_{mTOR}(t) + 3];$$

$$O_{GSK-3/APC}(t+1) = sgn[+O_{APC}(t) + O_{GSK-3}(t) - 1];$$

$$O_{beta-cat}(t+1) = sgn[-O_{GSK-3/APC}(t) - O_{p53}(t) + 1];$$

$$O_{Slug}(t+1) = sgn[-O_{p53/Mdm2}(t) + O_{NF-kB}(t) + O_{TCF}(t)];$$

$$O_{mTOR}(t+1) = sgn[+O_{RAGS}(t) + O_{AKT}(t) + O_{RHEB}(t) - O_{AMPK}(t) - 1];$$

$$O_{HIF1}(t+1) = sgn[+O_{Hypoxia}(t) + O_{mTOR}(t) - 2O_{VHL}(t) - O_{PHDs}(t) + O_{Myc/Max}(t) - O_{p53}(t) - O_{FOXO}(t) + 2];$$

$$O_{COX412}(t+1) = sgn[+O_{HIF1}(t)];$$

$$O_{VHL}(t+1) = sgn[-O_{Hypoxia}(t) - O_{ROS}(t) + 1];$$

$$O_{PHDs}(t+1) = sgn[-O_{Hypoxia}(t) + O_{ROS}(t) + 1];$$

$$O_{Myc/Max}(t+1) = sgn[-O_{TGFbeta}(t) + O_{Myc}(t) + O_{Max}(t) - O_{MXI1}(t) - O_{SmadE2F}(t) - 1];$$

$$O_{Myc}(t+1) = sgn[+O_{NF-kB}(t) + O_{ERK}(t) - O_{HIF1}(t) + O_{E2F}(t) + O_{FosJun}(t) + O_{TCF}(t) + O_{Gli}(t) - 1];$$

$$O_{Max}(t+1) = 1;$$

$$O_{MXI1}(t+1) = sgn[+O_{HIF1}(t)];$$

$$O_{TSC1/TSC2}(t+1) = sgn[-O_{RAF}(t) - O_{ERK}(t) - O_{p90}(t) - O_{AKT}(t) + O_{HIF1}(t) + O_{p53}(t) + O_{AMPK}(t) + 1];$$

$$O_{RHEB}(t+1) = sgn[-O_{TSC1/TSC2}(t) + 1];$$

$$O_{p53}(t+1) = sgn[+O_{HIF1}(t) - O_{Bcl-2}(t) - O_{Mdm2}(t) + O_{CHK1/2}(t) + 1];$$

$$O_{Bcl-2}(t+1) = sgn[+2O_{NF-kB}(t) - O_{p53}(t) - O_{BAX}(t) - O_{BAD}(t)];$$

$$O_{BAX}(t+1) = sgn[-O_{HIF1}(t) + O_{p53}(t) - O_{Bcl-2}(t) + O_{JNK}(t)];$$

$$O_{BAD}(t+1) = sgn[-O_{RAF}(t) - O_{p90}(t) - O_{AKT}(t) - O_{HIF1}(t) + 1];$$

$$O_{Bcl-XL}(t+1) = sgn[-O_{p53}(t) - O_{BAD}(t) + 1];$$

$$O_{Rb}(t+1) = sgn[-O_{CycA}(t) - O_{CycB}(t) - O_{CycD}(t) - O_{CycE}(t) - O_{Mdm2}(t) + 2];$$

$$O_{E2F}(t+1) = sgn[-2O_{Rb}(t) - O_{CycA}(t) - O_{CycB}(t) + O_{E2F}(t) + 1];$$

$$O_{p14}(t+1) = sgn[+O_{Ras}(t) + O_{Myc/Max}(t) + O_{E2F}(t) - 3];$$

$$O_{CycA}(t+1) = sgn[+O_{CycA}(t) - O_{Rb}(t) - O_{cdc20}(t) - O_{p27}(t) - O_{p21}(t) + O_{E2F/CyclinE}(t) + O_{cdh1/UbcH10}(t)];$$

$$O_{CycB}(t+1) = sgn[-O_{p53}(t) - O_{cdh1}(t) - O_{cdc20}(t) - O_{p27}(t) - O_{p21}(t) + 1];$$

$$O_{CycD}(t+1) = sgn[+O_{NF-kB}(t) - 2O_{GSK-3}(t) + O_{Myc/Max}(t) - O_{p27}(t) - O_{p21}(t) - O_{p15}(t) - O_{FOXO}(t) + O_{FosJun}(t) + O_{TCF}(t) + O_{Gli}(t)];$$

$$O_{CycE}(t+1) = sgn[-O_{Rb}(t) + O_{E2F}(t) - O_{CycA}(t) - O_{p27}(t) - O_{p21}(t)];$$

$$O_{cdh1}(t+1) = sgn[-O_{CycA}(t) - O_{CycB}(t) + O_{cdc20}(t) + 1];$$

$$O_{cdc20}(t+1) = sgn[O_{CycB}(t) - O_{cdh1}(t)];$$

$$O_{UbcH10}(t+1) = sgn[+O_{CycA}(t) + O_{CycB}(t) - O_{cdh1}(t) + O_{cdc20}(t) + O_{UbcH10}(t)];$$

$$O_{p27}(t+1) = sgn[-O_{AKT}(t) + O_{HIF1}(t) - O_{Myc/Max}(t) - O_{CycA}(t) - O_{CycB}(t) - O_{CycD}(t) + O_{SmadMiz-1}(t) + 1];$$

$$O_{p21}(t+1) = sgn[-O_{AKT}(t) + O_{HIF1}(t) - O_{Myc/Max}(t) + O_{p53}(t) + O_{SmadMiz-1}(t) - O_{hTERT}(t) + 1];$$

$$O_{Mdm2}(t+1) = sgn[+O_{AKT}(t) + O_{p53}(t) - O_{p14}(t) - O_{ATM/ATR}(t) + 1];$$

$$O_{Smad}(t+1) = sgn[O_{TNFalpha}(t) + O_{TGFbeta}(t)];$$

$$O_{SmadMiz-1}(t+1) = sgn[+O_{Smad}(t) + O_{Miz-1}(t) - 1];$$

$$O_{SmadE2F}(t+1) = sgn[+O_{Smad}(t)];$$

$$O_{p15}(t+1) = sgn[+O_{SmadMiz-1}(t) + O_{Miz-1}(t)];$$

$$O_{FADD}(t+1) = sgn[+O_{TNFalpha}(t)];$$

$$O_{Caspase8}(t+1) = sgn[+O_{FADD}(t)];$$

$$O_{Bak}(t+1) = sgn[+O_{Caspase8}(t)];$$

$$O_{JNK}(t+1) = sgn[+O_{TGFbeta}(t)];$$

$$O_{FOXO}(t+1) = sgn[-O_{AKT}(t) + 2];$$

$$O_{FosJun}(t+1) = sgn[+O_{ERK}(t) + O_{JNK}(t)];$$

$$O_{ROS}(t+1) = sgn[-O_{COX412}(t) - O_{GSH}(t)];$$

$$O_{AMPK}(t+1) = sgn[-O_{GFs}(t) + O_{AMP/ATP}(t) + O_{HIF1}(t) + O_{ATM/ATR}(t) + 1];$$

$$O_{Cytoc/APAF1}(t+1) = sgn[-O_{AKT}(t) + O_{p53}(t) - O_{Bcl-2}(t) + O_{BAX}(t) - O_{Bcl-XL}(t) + O_{Caspase8}(t) + O_{Bak}(t)];$$

$$O_{Caspase9}(t+1) = sgn[+O_{Cytoc/APAF1}(t)];$$

$$O_{Apoptosis}(t+1) = sgn[+O_{Caspase8}(t) + O_{Caspase9}(t)];$$

$$O_{E-cadh}(t+1) = sgn[-O_{NF-kB}(t) - O_{Slug}(t) - O_{Snail}(t) + 3];$$

$$O_{Glut-1}(t+1) = sgn[+O_{AKT}(t) + O_{HIF1}(t) + O_{Myc/Max}(t) - 1];$$

$$O_{hTERT}(t+1) = sgn[O_{NF1}(t) + O_{NF-kB}(t) + O_{AKT}(t) + O_{HIF1}(t) + O_{Myc/Max}(t) - O_{p53}(t) - O_{SmadMiz-1}(t) - O_{eEF2}(t) - 4];$$

$$O_{VEGF}(t+1) = sgn[+O_{HIF1}(t) + O_{Myc/Max}(t)];$$

$$O_{E2F/CyclinE}(t+1) = sgn[+O_{E2F}(t) + O_{CycE}(t) - 1];$$

$$O_{cdh1/UbcH10}(t+1) = sgn[+O_{cdh1}(t) + O_{UbcH10}(t) - 1];$$

$$O_{TAK1}(t+1) = sgn[+O_{TNFalpha}(t)];$$

$$O_{GSH}(t+1) = sgn[+O_{NF-kB}(t) + O_{Myc/Max}(t) + O_{p21}(t)];$$

$$O_{TCF}(t+1) = sgn[+O_{beta-cat}(t) - O_{TAK1}(t)];$$

$$O_{Miz-1}(t+1) = sgn[-O_{Myc/Max}(t) + 1];$$

$$O_{p70}(t+1) = sgn[+O_{PDK1}(t) + O_{mTOR}(t)];$$

$$O_{ATM/ATR}(t+1) = sgn[+O_{DnaDamage}(t)];$$

$$O_{CHK1/2}(t+1) = sgn[+O_{ATM/ATR}(t)];$$

$$O_{DNA\,Repair}(t+1) = sgn[+O_{ATM/ATR}(t)];$$

$$O_{eEF2K}(t+1) = sgn[+O_{p90}(t) + O_{p70}(t)];$$

$$O_{eEF2}(t+1) = sgn[-O_{eEF2K}(t) + 1];$$

$$O_{p53/PTEN}(t+1) = sgn[+O_{PTEN}(t) + O_{p53}(t) - 1];$$

$$O_{LDHA}(t+1) = sgn[+O_{HIF1}(t) + O_{Myc/Max}(t) - 1];$$

$$O_{AcidLactic}(t+1) = sgn[+O_{LDHA}(t)];$$

$$O_{Snail}(t+1) = sgn[+O_{NF-kB}(t) - O_{GSK-3}(t) - O_{p53}(t) + O_{Smad}(t) - 1];$$
