# Supplementary material for: Precritical State Transition Dynamics in the Attractor Landscape of a Molecular Interaction Network Underlying Colorectal Tumorigenesis
Source: PLoS One. 2015 Oct 6;10(10):e0140172. doi: 10.1371/journal.pone.0140172 (PMC4595005; doi:10.1371/journal.pone.0140172)
Supplement: S2 Text — (PDF) [file pone.0140172.s007.pdf]

S2 Text. Relative effects on the observed variation in the estimate of the basin size by the mutation, the noise, and the sampling procedure

The relative effects on the observed variation in the estimate of the basin size by the genetic perturbation (mutation), the non-genetic perturbation (noise), and the sampling procedure used in this study need to be discussed. S2(a) and (b) Fig. show the Euclidean distance [1] between two vectors of (B_A, B_P, B_Q) as a result of estimating the basin size twice in the conditions of N_I=0 during colorectal tumorigenesis (colorectal tumorigenesis is driven by the same sequence as the one in Fig. 2: No. 73 in S1 Table). S2(a) Fig. shows the results from two trials for the same sample size (trial 1 and trial 2 with 10,000 initial states in S3 Table) as follows:

$$M_{E1} = \sqrt{(B_{A0}^{T1} - B_{A0}^{T2})^2 + (B_{P0}^{T1} - B_{P0}^{T2})^2 + (B_{Q0}^{T1} - B_{Q0}^{T2})^2}\,, \quad (1)$$

where T1 and T2 represent the trial no. of the sampling procedure. S2(b) Fig. is derived from two different sample sizes (trial 1 with 10,000 initial states and trial 2 with 1,000,000 initial states in S3 Table) as follows:

$$M_{E2} = \sqrt{(B_{A0}^{G1} - B_{A0}^{G2})^2 + (B_{P0}^{G1} - B_{P0}^{G2})^2 + (B_{Q0}^{G1} - B_{Q0}^{G2})^2}\,, \quad (2)$$

where G1 and G2 indicate the two different sample sizes used in the sampling procedure. M_{E1} and M_{E2} indicate the variation in the estimate of the basin size by the sampling procedure itself, regardless of the genetic mutation and the noise. In Fig. 2(d), M_F actually represents the Euclidean distance between two vectors of (B_A, B_P, B_Q), as a result of the variance in the estimate of the basin size by the impact of noise and the sampling procedure. Nevertheless, as shown in S2 Fig., the effect on the variation in the estimate of the basin size by the sampling

procedure is negligible compared to the one by the genetic and non-genetic perturbations shown in Fig. 2(d). Thus, only the effects on the variation in the estimate of the basin size by genetic and non-genetic perturbations can be analyzed. This provides the rationale for the robustness of M_F regardless of the sampling procedure.

Reference

1. Stahl S. Geometry from Euclid to Knots: Courier Corporation; 2012.
